# NbBayesLM: bayesian prediction of nanobody thermostability using protein language model

**DOI:** 10.3389/fbinf.2026.1832968

**Published:** 2026-06-03

**Authors:** Fairuz Shadmani Shishir, Rokunuzjahan Rudro, Bishnu Sarker, Cuncong Zhong, Sumaiya Shomaji

**Affiliations:** 1 Department of Electrical Engineering and Computer Science, University of Kansas, Lawrence, KS, United States; 2 Department of Bioengineering in the University of Texas, Dallas, TX, United States; 3 Department of Information Science in the University of North Texas, Denton, TX, United States

**Keywords:** bayesian deep learning, bioinformatics, biomedical AI, nanobody engineering, nanobody thermostability, protein language models

## Abstract

Nanobodies, single-domain antibodies derived from camelids, are promising biologics due to their small size and high stability. Accurate prediction of their thermostability is critical for therapeutic and diagnostic applications. Due to their ability to bind conformationally constrained epitopes that are typically inaccessible to conventional antibodies, nanobodies represent a uniquely valuable modality in therapeutic target engagement and drug discovery. Existing methods for predicting nanobody thermostability often rely on limited data, handcrafted features, or black-box machine learning models that lack uncertainty quantification, limiting their generalizability and reliability. To address these gaps, this study, named NbBayesLM, proposes a Bayesian neural network (BNN) approach that integrates protein language model (PLM) embeddings with chemical property features to predict nanobody thermostability. In our formulation, physicochemical properties are incorporated as Bayesian priors, providing biologically meaningful constraints that guide posterior learning and improve model interpretability. Trained on a dataset of 10,630 nanobody sequences with experimentally determined $T_{m}$ values, our model achieves a mean absolute error of 1.89 °C and $R^{2}$ score of 0.67, outperforming existing models reported in the literature, while the fusion mechanism enhances performance over unimodal approaches and the BNN architecture provides well-calibrated uncertainty estimates to guide candidate selection and accelerate nanobody engineering.

## 1 Introduction

Nanobodies, or VHH domains, are single-domain antibodies derived from camelids, valued for their small size (12–15 kDa), high solubility, and inherent thermostability (Muyldermans, 2013). These properties make them ideal for applications in cancer therapy, diagnostics, and imaging (Muyldermans, 2021). Nanobody thermal stability (Kunz et al., 2018), characterized by melting temperature $\left(T_{m}\right)$ , is a fundamental property that governs nanobody function, therapeutic efficacy, and biotechnological applications. The melting temperature represents the temperature at which half of the nanobody molecules unfold, making it a critical parameter for understanding nanobody stability under physiological and storage conditions. Accurate prediction of nanobody melting temperatures has profound implications across multiple domains, from therapeutic antibody development and drug formulation to understanding nanobody engineering strategies and shelf-life optimization (Kunz et al., 2018). Traditional approaches to melting temperature prediction have predominantly relied on physicochemical features derived from amino acid sequences, including hydrophobicity, charge distribution, secondary structure propensities, and other related sequence-based characteristics (Khodabakhsh et al., 2018). While these methods have provided valuable insights into the determinants of nanobody stability, they often fall short of capturing the complex, context-dependent relationships that govern thermal stability in these unique single-domain antibodies (Salvador et al., 2019). The limitation stems from their inability to account for the specific structural constraints of nanobodies, including their characteristic disulfide bonds, complementarity-determining region (CDR) flexibility, and the unique folding patterns that distinguish them from conventional antibodies.

Recent advances in protein language models, particularly the Evolutionary Scale Modeling (ESM) family of transformers (Lin et al., 2023), have revolutionized our understanding of protein sequences by learning rich, contextualized representations from millions of evolutionary sequences. These models capture evolutionary information that reflects the natural selection pressures on antibody domains, encoding structural and functional constraints that are not readily apparent from physicochemical properties alone. The ESM-2 model, trained on over 65 million protein sequences including extensive antibody repertoires, has demonstrated remarkable success in various protein prediction tasks, suggesting its potential for nanobody thermal stability prediction. However, existing approaches to nanobody property prediction often suffer from two critical limitations. First, they typically provide point estimates without quantifying prediction uncertainty, which is essential for therapeutic applications where decision-making requires confidence assessment and risk management. Second, most methods fail to effectively integrate complementary information sources, such as evolutionary embeddings and physicochemical features, in a principled manner that accounts for their relative contributions and interactions specific to nanobody structural biology. Bayesian neural networks offer a natural solution to these challenges by providing principled uncertainty quantification and enabling robust integration of heterogeneous feature types (Goan and Fookes, 2020). By modeling weight distributions rather than point estimates (Rosenblueth, 1975), Bayesian approaches can distinguish between epistemic uncertainty (model uncertainty due to limited data) and aleatoric uncertainty (inherent data noise), providing valuable insights into prediction reliability (Gal and Ghahramani, 2016). This uncertainty decomposition is particularly crucial for nanobody engineering applications, where understanding prediction confidence can guide experimental design, therapeutic candidate selection, and formulation development strategies.

In this work, we present a Bayesian Fusion Model that addresses these limitations by combining evolutionary information from ESM-2 transformers with physicochemical features through a principled Bayesian framework. Our approach leverages multi-head attention mechanisms to capture long-range dependencies within evolutionary embeddings while employing Bayesian linear layers to model parameter uncertainty and enable robust feature fusion. The model provides not only accurate melting temperature predictions but also quantifies both epistemic and aleatoric uncertainties, offering a comprehensive assessment of prediction reliability for nanobody thermal stability.

The key contributions of this work are as follows:

- To the best of our knowledge, we introduce the first application of a Bayesian neural network framework that integrates evolutionary sequence representations from ESM-2 with physicochemical descriptors through attention-based fusion for nanobody thermostability prediction.
- We explicitly decompose predictive uncertainty into epistemic and aleatoric components, enabling principled confidence estimation for downstream decision-making in nanobody development.
- Through rigorous 5-fold cross-validation, we demonstrate that the proposed method consistently outperforms state-of-the-art baselines and yields well-calibrated uncertainty estimates, thereby enhancing its reliability and applicability in therapeutic nanobody engineering.

Our results show that the fusion of evolutionary and physicochemical features, combined with Bayesian uncertainty quantification, represents a significant advancement in computational approaches to nanobody thermal stability prediction. The remainder of this paper is organized as follows: Section 2 provides the background, Section 3 describes the methodology, Section 4 presents the experimental design and results, Section 5 offers a discussion of the findings ad limitations, and Section 6 concludes the paper.

## 2 Related work

Camelids produce heavy-chain-only antibodies whose isolated variable domains (VHHs, or nanobodies) condense full antigen-binding capacity into a 12–15 kDa scaffold, far smaller than IgG (Hamers-Casterman et al., 1993). Their monomeric, soluble architecture penetrates dense tissues, supports intracellular expression, and, when formatted appropriately, can cross the blood–brain barrier or neutralize pathogens such as SARS-CoV-2 (Pavan et al., 2023). To advance as drugs, nanobodies must remain folded across fermentation, purification, storage, and delivery. Melting temperature $\left(T_{m}\right)$ is widely used as a proxy for developability: higher $T_{m}$ correlates with reduced aggregation, longer shelf life, and protease resistance. While nano-DSF (Magnusson et al., 2019) enables higher-throughput screening than differential scanning calorimetry, it still cannot match the scale of modern display libraries ( $10^{5}$ variants) (Bruxvoort et al., 2020). NbThermo, the first nanobody-specific stability repository, provides ∼500 curated $T_{m}$ records with metadata, yet remains sparse for large-scale modeling (Huang and Lin, 2024).

Early regressors based on hand-crafted features [e.g., PROTS-RF (Li and Fang, 2012)] rarely exceeded $R^{2}\approx 0.6$ and offered no mechanism for uncertainty estimation (Li and Fang, 2012). Self-supervised transformers capture evolutionary and structural constraints at scale (Elnaggar et al., 2021). TEMPRO fuses ESM-2 embeddings, NetSurfP-3 residue accessibility, and AlphaFold2 pLDDT scores, reducing mean absolute error to ∼4 °C on NbThermo (Alvarez and Dean, 2024; Høie et al., 2022; Jumper et al., 2021; Wang et al., 2024). TemBERTure further fine-tunes a BERT-style model on 1.2 M sequences, achieving state-of-the-art accuracy across mesophilic and extremophilic subsets with attention-based interpretability (Wang et al., 2024). Yet such PLM-driven regressors remain deterministic, providing only point estimates without conveying prediction reliability. This limitation is critical in prospective design, where every assay is costly and labels themselves carry noise (e.g., buffer or scan-rate effects).

Bayesian frameworks directly address these challenges by providing (1) uncertainty quantification and (2) sample-efficient exploration of sequence space. BayeStab couples graph features with Concrete-Dropout inference to yield calibrated intervals and noise-floor estimates across stability datasets (Wang and Tang, 2022). RESP2 extends this with variational Bayesian networks disentangling epistemic and aleatoric error (Garcia et al., 2024), and benchmarking confirms that calibrated models better guide experimental priorities when false positives are costly (Ryu et al., 2019). AntBO applies Bayesian optimization in CDRH3 space, doubling hit rates with <1% of the naïve screening budget (Khan et al., 2023). Related acquisition strategies have delivered high-success scFv libraries (Parkinson et al., 2025) and active-learning loops coupling free-energy calculations with Bayesian acquisition for mutation ranking (Patel et al., 2024). Table 1 summarizes representative models from existing literature that focus on protein thermostability prediction and nanobody design.

**TABLE 1:** Representative models for thermostability prediction and antibody design.

Model	Purpose/Output	Principal dataset(s)	Limitations
TEMPRO (Alvarez and Dean, 2024)	Nanobody $T_{\text{m}}$ regression using PLM + structural features	NbThermo (n≈500)	Small dataset; nanobody-specific; requires 3D structure
TemBERTure (Wang et al., 2024)	$T_{\text{m}}$ regression and thermophile classification with attention maps	TemBERTureDB^1^ ( ∼0.6 M seqs)	Limited experimental validation; potential domain bias
PROTS-RF (Li and Fang, 2012)	$\Delta T_{\text{m}}$ / ΔΔG for point mutations (random forest)	ProThermDB ( ∼32 k mutants)	Single mutations only; limited feature engineering
BayeStab (Wang and Tang, 2022)	Bayesian GNN + concrete-dropout; calibrated uncertainty for ΔΔG	ProTherm subsets S2648, S350, S611*etc.*	Requires graph construction; computational complexity
RESP2 (Garcia et al., 2024)	Variational bayesian multitask network (stability + other properties)	Mixed ProTherm + solubility/pH panels	Multi-task trade-offs; limited interpretability
AntBO (Khan et al., 2023)	Combinatorial bayesian optimisation of CDRH3 sequences (affinity + developability)	OAS repertoires; 192-variant wet-lab set	CDRH3 focus only; limited experimental scope

## 3 Methodology

Nanobody sequences pose challenges for machine learning due to their variable lengths and intricate residue dependencies (Asaadi et al., 2021). Transformers, developed by (Vaswani et al., 2017), with their parallel processing and self-attention mechanisms, address these issues effectively. Unlike sequential models, Transformers capture long-range dependencies through scaled dot-product attention, which operates on queries (Q), keys (K), and values (V). As shown in Equation 1, attention weights are computed by the normalized similarity between Q and K, scaled by $d_{k}$ , and then applied to V. Multi-head attention further extends this mechanism by computing multiple attention heads in parallel and concatenating their outputs, as defined in Equation 2. Each individual head is obtained by linearly projecting Q, K, and V and applying the attention operation, as shown in Equation 3.

$$\text{Attention}\left(Q,K,V\right)=\text{softmax}\left(\frac{QK^{T}}{\sqrt{d_{k}}}\right)V$$ (1)

$$\text{MultiHead}\left(Q,K,V\right)=\text{Concat}\left({\text{head}}_{1},\ldots ,{\text{head}}_{h}\right)W^{Q}$$ (2)

$${\text{head}}_{i}=\text{Attention}\left(QW_{i}^{Q},KW_{i}^{K},VW_{i}^{V}\right)$$ (3)

Advances such as AlphaFold (Jumper et al., 2021) and AlphaFold 3 (Abramson et al., 2024) highlight the power of deep learning in modeling complex biological systems. Building on these advances, protein language models (PLMs) apply the same self-supervised learning principles to the “language” encoded by amino acid sequences. By training on millions of unlabeled proteins, PLMs such as ESM (Lin et al., 2023), AbLang (Olsen et al., 2022), and ESMFold (Lin et al., 2022) learn to predict masked residues or next-token likelihoods, thereby internalizing evolutionary and biophysical constraints. The resulting fixed-length embeddings condense each sequence into a vector that captures local motifs, global folding tendencies, and residue co-evolution patterns—all without requiring multiple sequence alignments or explicit structural input. These learned representations have proven highly effective for downstream tasks, from mutation effect prediction to functional annotation, and are especially well-suited for regression tasks like thermostability estimation because they embed both sequence context and implicit structural signals.

Evolutionary Scale Modeling (ESM) (Lin et al., 2023) epitomizes the power of large-scale transformer training on protein data. Pretrained on over 65 million sequences, ESM’s attention layers can discern long-range residue interactions that underlie stability, catalysis, and binding. When fine-tuned on melting temperature measurements, ESM embeddings enable rapid, accurate regression models that predict thermostability from a single input sequence in milliseconds—far outpacing structure-based simulations. Moreover, because ESM does not rely on curated alignments, it excels on novel or orphan proteins where homologous templates are lacking. AbLang (Olsen et al., 2022) and ESMFold (Lin et al., 2022) extend these ideas in complementary directions. AbLang specializes in antibodies by training exclusively on tens of millions of immunoglobulin sequences; its masked-reconstruction objective yields embeddings that spotlight hypervariable regions and framework residues critical for thermal resilience. This domain focus delivers a 15%–20% boost in thermostability-prediction accuracy for antibody engineering tasks.

**FIGURE 1:**
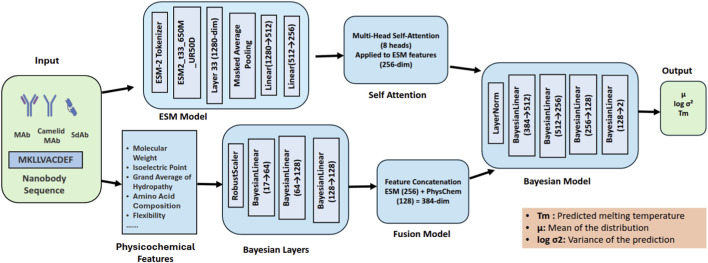
Pipeline overview for bayesian nanobody thermostability model (NbBayesLM).

ESMFold, by contrast, couples ESM embeddings with a lightweight structural module to predict 3D coordinates directly from sequence. This hybrid approach combines the speed and simplicity of embedding-based methods with the atomic-level insights of structural modeling—producing high-throughput stability assessments that capture hydrophobic core packing, disulfide patterns, and salt-bridge networks, all without the multi-hour runtimes or alignment dependencies of traditional structure predictors like AlphaFold 2. Building on the transformer revolution in protein modeling, ProtBERT (Brandes et al., 2022) applies the BERT architecture directly to amino acid sequences, treating each residue as a “token” in a contiguous string. By pretraining on large corpora of protein data—often drawn from UniRef50 or UniRef100—ProtBERT learns to predict masked residues within each sequence, forcing its deep attention layers to capture both local motif patterns and long-range interactions that underlie folding and function. In practice, this means that during pretraining ProtBERT internalizes contextual relationships between amino acids: for instance, when a hydrophobic residue at one position frequently co-occurs with another at a distant site, the model’s attention heads learn to strengthen that connection. Figure 1 summarizes our methodology pipeline.

### 3.1 Chemical property features

To complement the contextual information encoded by the ESM-2 embeddings, we extract a 17-dimensional vector of classic physicochemical descriptors for every nanobody sequence. Features are computed with Biopython 1.83s ProteinAnalysis class (Cock et al., 2009) and residue indices from AAindex (Kawashima et al., 2007). We choose descriptors that earlier experimental and *in silico* work has linked to thermal stability, solubility, or backbone rigidity (Potapov et al., 2009; Li et al., 2023; Lee and Wang, 2024). Each feature is standardized (zero mean, unit variance) on the training set before concatenation with the PLM vector. Table 2 summarizes the physicochemical features used for the study.

**TABLE 2:** Physicochemical descriptors.

Sym	Definition	Thermostability rationale
SeqLen	Number of residues	Domain size vs. folding cooperativity
Mw	Molecular weight (Da)	Larger hydrophobic cores
pI	Isoelectric point	pH-dependent electrostatics
NetQ	Net charge at pH 7.4	Colloidal stability
II	Instability index	<40 ⇒ *in-vivo* stability
GRAVY	Grand average hydropathy	Core packing strength
AliphIdx	Aliphatic index	Hydrophobic volume
Aro	Aromaticity fraction	π – π stacking
Flexµ	Mean karplus–Schulz flexibility	Lower mobility $↑T_{m}$
Helix	Helix fraction	H-bond network balance
Turn	Turn fraction	Loop entropy contribution
Sheet	β -sheet fraction	Inter-strand H-bonding
HydroFrac	Hydrophobic residue fraction	Core stability
PolarFrac	Polar residue fraction	Surface hydration
ChargedFrac	Charged residue fraction	Salt-bridge networks
AromFrac	F/Y/W fraction	π -stacking, cation– π contacts
CysFrac	Cysteine fraction	Disulfide crosslinks
ProFrac	Proline fraction	Lowers unfolding entropy
GlyFrac	Glycine fraction	Backbone flexibility (often lowers $T_{m}$ )

### 3.2 Feature fusion

To integrate the PLM embeddings and chemical property features, we employed a concatenation-based fusion strategy (Dai et al., 2021). The 1280-dimensional PLM embedding vector was concatenated with the 17-dimensional chemical property vector, resulting in a 1297-dimensional input vector for each nanobody. To address potential scale mismatches, both feature sets were normalized using z-scores computed on the training set. We also explored an alternative fusion approach using a multi-modal neural network with separate branches for PLM and chemical features, merged *via* a dense layer with attention weighting. However, preliminary experiments indicated that concatenation provided comparable performance with lower computational complexity, so it was selected for the final model.

### 3.3 Bayesian neural network design

We developed a Bayesian ESM Fusion Model that combines evolutionary and physicochemical information through a principled Bayesian framework (Blundell et al., 2015; Tran et al., 2019). The architecture consists of several key components: ESM Feature Processing Branch: ESM Features→LayerNorm→ Linear(1280→512)→ReLU→Dropout(0.1)→Linear(512→256) Physicochemical Feature Branch: PhysChem Features→ LayerNorm→BayesianLinear(17→64)→ReLU→BayesianLinear (64→128)→BayesianLinear(128→128) Attention-Based Fusion: We employed multi-head self-attention (8 heads) on the processed ESM features to capture long-range dependencies within the evolutionary embeddings. Fusion Network: The attended ESM features and physicochemical embeddings were concatenated and passed through a Bayesian multi-layer perceptron: Concat(256,128)→BayesianLinear(384→512)→ BayesianLinear(512→256)→BayesianLinear(256→128)→ BayesianLinear(128→2) . The final layer outputs both mean and log-variance predictions to model aleatoric uncertainty.

#### 3.3.1 Bayesian layers

Each Bayesian linear layer parameterizes the weight and bias distributions using the reparameterization trick:

$$w∼N\left(\mu_{w},\sigma_{w}^{2}\right),\;\sigma_{w}=\text{softplus}\left(\rho_{w}\right)+\epsilon$$ (4)

where $\mu_{w}$ and $\rho_{w}$ are learnable parameters, and $\epsilon =10^{-6}$ is added to ensure numerical stability during training. Equation 4 allows the model to learn both the mean and uncertainty of each weight parameter while maintaining a positive standard deviation.

#### 3.3.2 Training loss

We employed a composite loss function combining negative log-likelihood and KL divergence, motivated by (Kingma and Welling, 2013):

$$L=E_{q\left(w\right)}\left[\log p\left(y|x,w\right)\right]+\beta \cdot KL\left[q\left(w\right)\|\;p\left(w\right)\right]$$ (5)

where:

- The likelihood term models heteroscedastic uncertainty: $p\left(y|x,w\right)=N\left(y;\mu \left(x,w\right),\exp\left(\sigma^{2}\left(x,w\right)\right)\right)$

- The KL term regularizes the posterior toward the prior: $p\left(w\right)=N\left(0,0.5^{2}\right)$

-
β follows a cyclical schedule proposed by (Fu et al., 2019)

Equation 5 therefore balances predictive accuracy with uncertainty-aware posterior learning.

We performed uncertainty quantification through Monte Carlo sampling with N=50 forward passes during inference, leveraging both dropout and Bayesian weight sampling.

#### 3.3.3 Uncertainty decomposition

Total predictive uncertainty was decomposed into:

$$\sigma_{\text{epistemic}}^{2}=\text{Var}\;\left[E\left[y|x,w\right]\right]$$ (6)

$$\sigma_{\text{aleatoric}}^{2}=E\;\left[\text{Var}\left[y|x,w\right]\right]$$ (7)

$$\sigma_{\text{total}}^{2}=\sigma_{\text{epistemic}}^{2}+\sigma_{\text{aleatoric}}^{2}$$ (8)

Equation 6 quantifies uncertainty arising from limited model knowledge, whereas Equation 7 captures irreducible uncertainty inherent in the data acquisition process. Equation 8 provides the overall predictive uncertainty used during inference and uncertainty-aware evaluation.

## 4 Experimental design and results analysis

We adopted the data in the NanoMelt study (Barkai and Linder, 2023), incorporating a significantly larger and more diverse set of nanobody (VHH) sequences to support robust machine learning analyses. The original NanoMelt protocol, detailed in its supplementary materials, was briefly stated here to ensure methodological consistency. The labeled thermostability dataset consists of 640 non-redundant nanobody sequences with experimentally measured melting temperatures $\left(T_{\text{m}}\right)$ . A total of 511 sequences were curated from peer-reviewed publications and the NbThermo database (Huang and Lin, 2024), while 129 sequences were newly characterized using intrinsic-fluorescence nanoDSF. These assays were conducted under standardized conditions, 20 mM HEPES, 150 mM NaCl, pH 7.4, with a uniform heating rate of 2 °C ${\text{min}}^{-1}$ . The raw fluorescence signals (330 nm and 350 nm) were analyzed using a two-state unfolding model to derive consistent $T_{\text{m}}$ values. Sequences with 100% identity were collapsed using CD-HIT (Fu et al., 2012), and non-canonical amino acids were removed. In cases of multiple experimental assays for the same sequence, a mean $T_{\text{m}}$ value was computed, provided the absolute deviation did not exceed 2.4 °C. The unlabeled nanobody corpus was computationally labeled using the CamSol (Sormanni et al., 2015) solubility prediction tool to generate Tm estimates. After removing duplicate sequences, our final training set consisted of 10,630 unique nanobody sequences. We carefully verified that no data leakage occurred between training and test sets by confirming that all test sequences were completely absent from the training data, ensuring valid performance evaluation.

### 4.1 Correlation and descriptive statistics

**FIGURE 2:**
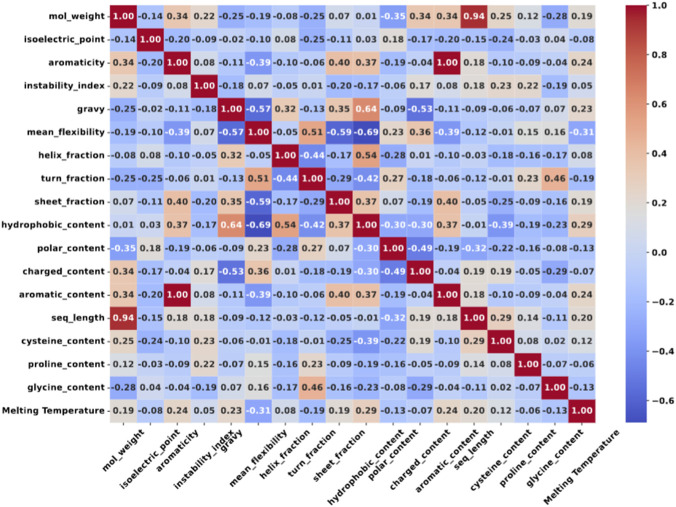
Pearson correlation heatmap between all physicochemical features with respect to melting temperature.

We examined the relationships between physicochemical descriptors including molecular weight, isoelectric point, aromaticity, instability index, GRAVY score, mean flexibility, secondary structure fractions (helix, turn, sheet), amino acid composition (hydrophobic, polar, charged, aromatic, cysteine, proline, glycine content), sequence length, and melting temperature through Pearson correlation analysis. The correlation matrix was visualized as a heatmap in Figure 2, where color intensity reflects correlation strength and darker red tones represent stronger positive correlations, while blue tones indicate negative correlations between descriptor pairs.

To broaden the sequence diversity and support representation learning, the study (Barkai and Linder, 2023) compiled an unlabeled corpus of 10,000 unique VHH sequences from five publicly available repositories: (1) the camelid subset of Observed Antibody Space (OAS) (Kovaltsuk et al., 2018), (2) the Wally structural benchmark (Munro and Singh, 2020), (3) deep mutational scanning datasets from the Marks Laboratory (Munro and Singh, 2020), (4) all annotated VHH chains available in the Protein Data Bank (PDB) (Burley et al., 2017), and (5) an additional set of 1,000 recently released sequences under permissive licenses. All signal peptides were removed, and sequences were clustered at 100% identity to eliminate duplicates, which were the inputs used in this experiment. Any sequence overlapping with the curated NanoMelt dataset was excluded. The resulting dataset was aligned using the MAFFT L-INS-i algorithm to facilitate downstream analyses. After removing the duplicate values and merging, the final dataset comprises 10,630 unique VHH sequences, of which 630 are labeled with high-quality experimental $T_{\text{m}}$ values.

### 4.2 External test data

An external test set comprising 77 single-domain antibodies (sdAbs) with experimentally determined melting temperatures ranging from 46 ° C to 85.51 ° C was assembled to evaluate the performance of thermal stability prediction models (Alvarez and Dean, 2024; Gómez-Mulas et al., 2025b; Gómez-Mulas et al., 2025a). To ensure rigorous validation, the dataset was curated to avoid any overlap with the training set. The melting temperatures were obtained using a variety of experimental techniques, including circular dichroism (CD), differential scanning fluorimetry (DSF), nano differential scanning fluorimetry (nanoDSF), and differential scanning calorimetry (DSC).

### 4.3 Feature preprocessing

Physicochemical features were standardized using the RobustScaler implementation from the scikit-learn package (Pedregosa et al., 2011), which scales features according to the interquartile range (IQR), making it more resilient to outliers compared to standard normalization techniques such as StandardScaler or min-max scaling. This preprocessing step ensures that features with skewed distributions, such as molecular weight or isoelectric point, do not disproportionately influence the model. The ESM-2 embeddings, derived from the pretrained protein language model, were used without further normalization, as these embeddings are inherently scaled and optimized during model pretraining. Given their high dimensionality (e.g., 1,280 dimensions), no dimensionality reduction techniques (e.g., PCA (Maćkiewicz and Ratajczak, 1993) or t-SNE (Maaten and Hinton, 2008)) were applied in this study to preserve the full informational content of the sequence representations. Preliminary tests indicated that retaining the full embedding space contributed positively to model performance. All features were concatenated into a unified representation vector prior to model training. To avoid data leakage, the scaling parameters were fitted exclusively on the training data and subsequently applied to the validation and test sets. This pipeline was implemented using the scikit-learn preprocessing framework and integrated within a cross-validation routine to ensure consistency and reproducibility across folds.

### 4.4 Experimental setup

To rigorously evaluate the proposed Bayesian fusion framework, we employed the AdamW optimizer (Loshchilov and Hutter, 2017) with differentiated learning rates ( $1\times 10^{-5}$ for the ESM processor and $1\times 10^{-4}$ for the physicochemical branch and fusion MLP), weight decay of $1\times 10^{-5}$ , and gradient clipping at a maximum norm of 1.0. Training was conducted with a batch size of 4 (constrained by GPU memory for ESM-2 inference) for up to 80 epochs, with early stopping after eight epochs of non-improvement in validation $R^{2}$ , and learning rates were adaptively reduced using a ReduceLROnPlateau scheduler (Thakur et al., 2024) (factor 0.5, patience 3). To improve robustness, Gaussian noise (σ=0.01) was added to physicochemical features with a probability of 10% during training. Experiments were performed on an NVIDIA RTX 3090 GPU with CUDA support and 16 GB RAM, where ESM-2 inference required approximately 2.5 GB of GPU memory and the Bayesian fusion model added 100 MB; each fold required 30–45 min of training. The implementation was based on PyTorch for model development, scikit-learn for cross-validation, and BioPython’s ProtParam module (Cock et al., 2009) for protein feature computation.

### 4.5 Results analysis

As summarized in Table 3, the comprehensive performance evaluation across protein language models (PLMs) and traditional machine learning algorithms revealed distinct patterns that illuminate the effectiveness of different molecular representation strategies. All models were evaluated using rigorous 5-fold cross-validation (KFold, n=5 ) with shuffling and a fixed random seed to ensure consistent fold assignments and reproducible results. Among the traditional machine learning approaches, the combination of ESM embeddings with Lasso regression achieved the highest predictive accuracy, establishing the performance benchmark for this prediction task with a test $R^{2}$ of 0.62, mean squared error (MSE) of 8.56, and mean absolute error (MAE) of 2.18. Notably, Lasso regression demonstrated consistent superiority over other regression algorithms regardless of the underlying protein language model employed, suggesting that $L_{1}$ regularization effectively addresses the high-dimensional nature of protein embeddings. Sequence-based models, particularly ESM and ProtBERT, demonstrated superior predictive capabilities compared to structure-based approaches. ESM consistently ranked highest across all performance metrics, while ProtBERT showed comparable but slightly lower performance levels. AbLang presented a particularly interesting case study in embedding sensitivity. When paired with linear and ridge regression, AbLang embeddings yielded negative $R^{2}$ values; however, the application of Lasso regression and XGBoost resulted in substantial performance improvements, suggesting that the propensity of AbLang embeddings to overfit can be effectively controlled through appropriate regularization strategies. ProtBERT exhibited similar behavioral patterns, with Lasso regression providing optimal results and XGBoost achieving moderate performance levels.

**TABLE 3:** Performance of traditional ML models using different embedding models.

Embedding	Model	CV $R^{2}$	CV MSE	CV MAE	Test $R^{2}$	Test MSE	Test MAE
ESM	Linear regression	0.340	14.096	2.729	0.338	15.012	2.780
	Ridge regression	0.376	13.352	2.658	0.338	15.012	2.779
	Lasso regression	0.610	8.337	2.142	0.622	8.558	2.176
	XGBoost regressor	0.447	11.853	2.578	0.448	12.517	2.600
AbLang	Linear regression	−15.842	358.441	13.642	−2.201	72.639	6.141
	Ridge regression	−9.532	224.191	10.831	−2.116	70.711	6.061
	Lasso regression	0.558	9.449	2.242	0.543	10.347	2.341
	XGBoost regressor	0.465	11.449	2.529	0.485	11.680	2.558
ProtBERT	Linear regression	0.178	17.564	3.008	0.294	16.014	2.891
	Ridge regression	0.178	17.560	3.007	0.294	16.014	2.891
	Lasso regression	0.475	11.234	2.511	0.470	12.010	2.577
	XGBoost regressor	0.399	12.883	2.710	0.420	13.146	2.746
ESMFold	Linear regression	0.303	14.920	2.893	0.307	15.716	2.966
	Ridge regression	0.303	14.920	2.893	0.307	15.716	2.966
	Lasso regression	0.303	14.915	2.891	0.307	15.704	2.962
	XGBoost regressor	0.292	15.177	2.952	0.333	15.111	2.957

ESM model embeddings provide superior performance in comparison to the other PLMs.

In contrast, ESMFold demonstrated consistently modest performance across all algorithmic approaches ( $R^{2}\approx 0.30$ –0.33), indicating that structural embeddings do not confer substantial predictive advantages over sequence-based representations for this specific prediction task. This finding suggests that sequence information alone captures the essential features required for accurate prediction in our experimental context. Finally, the strong alignment between cross-validation and independent test set performance across the majority of model combinations provides robust evidence of generalization capability without significant overfitting. This consistency supports the robustness of our cross-validation framework and suggests that the observed performance differences are attributable to intrinsic model capabilities rather than dataset-specific biases.

#### 4.5.1 Bayesian fusion model performance

The Bayesian fusion model demonstrated substantially superior performance compared to traditional machine learning approaches across all evaluated metrics (Table 4). The model achieved an $R^{2}$ score of 0.67±0.0177 , representing a statistically significant improvement over the best-performing traditional model (Lasso regression with ESM embeddings, $R^{2}=0.622$ ). Concurrently, the Bayesian approach maintained lower error rates with MAE of 1.89±0.0765 and root mean squared error (RMSE) of 2.69±0.1015 , compared to traditional models that exhibited MAE values ranging from 2.176 to 13.642 and MSE values from 8.558 to over 70 for poorly performing configurations.

**TABLE 4:** Bayesian fusion model performance and uncertainty metrics.

Metric	ESM model (mean ± std)	ESM finetuned (mean ± std)
MAE	1.89 ± 0.0765	1.8166 ± 0.1033
RMSE	2.69 ± 0.1015	2.5156 ± 0.1319
$R^{2}$ score	0.67 ± 0.0177	0.7081 ± 0.0298
Epistemic uncertainty	11.18 ± 0.1639	12.9469 ± 0.3199
Aleatoric uncertainty	12.76 ± 0.6597	17.4219 ± 1.0575
Total uncertainty	23.94 ± 0.7802	30.3688 ± 1.3774

Beyond superior predictive accuracy, the Bayesian fusion model provides explicit uncertainty quantification capabilities entirely absent from traditional machine learning approaches. The model quantifies epistemic uncertainty at 11.18±0.1639 , aleatoric uncertainty at 12.76±0.6597 , and total uncertainty at 23.94±0.7802 , providing valuable insights into prediction confidence and model reliability.

**FIGURE 3:**
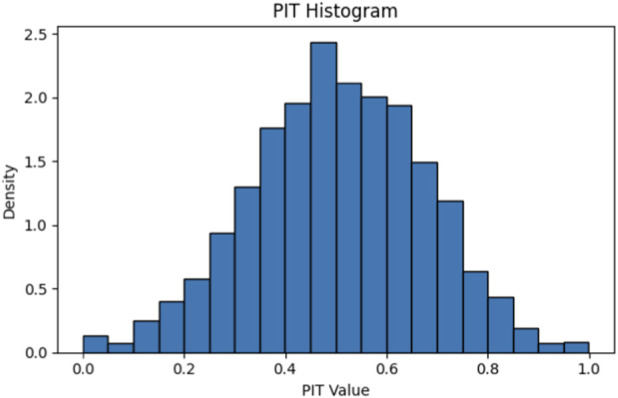
Probability Integral Transform (PIT) histogram of the predicted distributions.

Furthermore, the Bayesian model exhibited remarkable consistency across all performance metrics, as evidenced by consistently low standard deviations. Furthermore, to assess the contribution of domain adaptation, we fine-tuned ESM-2 on a curated corpus of nanobody sequences from our dataset and evaluated the resulting model under identical conditions. As shown in Table 4, fine-tuning yields modest improvements in predictive performance, MAE decreases from 1.89±0.08 to 1.82±0.10 , RMSE from 2.69±0.10 to 2.52±0.13 , and $R^{2}$ improves from 0.67±0.02 to 0.71±0.03 , suggesting that domain adaptation provides a marginal but consistent benefit to point estimation. However, fine-tuning substantially increases both epistemic and aleatoric uncertainty (epistemic: 11.18±0.16→12.95±0.32 ; aleatoric: 12.76±0.66→17.42±1.06 ), raising total predictive uncertainty from 23.94±0.78 to 30.37±1.38 . This elevation in uncertainty is noteworthy; while the fine-tuned encoder produces marginally better mean predictions, it introduces greater instability in the Bayesian weight posteriors, which may reflect overfitting to the relatively small nanobody fine-tuning corpus or distribution shift between the fine-tuning and evaluation sets. We therefore retain the pre-trained ESM-2 encoder in our final architecture, as it offers a more favourable calibration trade-off. This stability contrasts sharply with the high variability and sensitivity to embedding choice observed in both approaches, establishing the Bayesian fusion methodology as a more robust and informative modeling framework for our prediction tasks. Since the interpretability is crucial in deep learning models, shapley additive explanations (SHAP) analysis in Figure 3 reveals that cysteine fraction is the strongest driver of predicted $T_{m}$ , with high values increasing model output by nearly +2 °C, consistent with the thermostabilizing role of disulfide bonds in protein tertiary structure. Polar amino acid fraction and molecular weight follow as the next most influential features, each exhibiting broad bidirectional SHAP distributions ( ± 1.6–1.8), while helix and sheet fraction contribute positively at high values, reflecting the well-established relationship between ordered secondary structure and thermal stability. The instability index behaves intuitively, with high values suppressing predicted $T_{m}$ . Lower-ranked features, including GRAVY, aromaticity, and charged amino acid fraction, show modest but non-negligible contributions, whereas proline fraction is effectively negligible, suggesting limited discriminative power within this model. This structure-aware interpretation demonstrates that the model captures biologically meaningful determinants of nanobody thermostability. The code for this study is publicly available at https://github.com/FairuzShadmaniShishir/NanobodyThermostabilityPrediction.

### 4.6 Comparison with state-of-the-art methods

The proposed Bayesian model was benchmarked against Nanomelt, a widely adopted method for protein thermal stability prediction, using an external test set to ensure unbiased evaluation. As shown in Table 5, the Bayesian approach demonstrated substantial improvements across all performance metrics, establishing its superiority over the current state-of-the-art. The predictive accuracy improvements were most evident in the error metrics. The Bayesian model achieved a Mean Absolute Error (MAE) of 6.164 compared to Nanomelt’s 6.440, representing a 4.3% reduction in prediction error. More notably, the Root Mean Squared Error (RMSE) decreased from 8.320 to 7.726, indicating that the Bayesian model not only reduces average errors but also demonstrates superior performance on challenging cases with larger prediction errors. The explained variance comparison revealed the most significant advancement, with the Bayesian model achieving an $R^{2}$ score of 0.214 compared to Nanomelt’s 0.089. This 2.4-fold improvement in explained variance demonstrates that the Bayesian approach captures substantially more of the underlying structure-stability relationships, moving beyond simple pattern matching to a more mechanistic understanding. The model underperforms on external sequences exhibiting high local sequence variability within complementarity-determining regions (CDRs), despite overall framework conservation. These mutations often induce nonlinear effects on protein stability that are not fully captured by global physicochemical descriptors. Furthermore, elevated epistemic uncertainty in these regions indicates limited representation of such variants in the training data, while increased aleatoric uncertainty reflects inconsistencies introduced by heterogeneous experimental techniques (e.g., CD, DSC, DSF). This suggests that predictive uncertainty can effectively disentangle model limitations from intrinsic data noise. Finally, correlation analysis further reinforced the superior predictive capability of the Bayesian model. Both Pearson (0.584 vs. 0.487) and Spearman (0.535 vs. 0.443) correlation coefficients showed marked improvements over Nanomelt, with all correlations achieving statistical significance (p<0.001) . The higher Spearman correlation particularly indicates that the Bayesian model better captures non-linear relationships inherent in protein stability data. These comprehensive improvements across complementary metrics establish the Bayesian fusion approach as a significant advancement over existing methods, offering enhanced accuracy, robustness, and mechanistic insight for protein thermal stability prediction.

**TABLE 5:** Performance comparison of nanomelt and proposed model on external test set.

Metric	Nanomelt	NbBayesLM (proposed)
MAE ↓	6.440	6.164
RMSE ↓	8.320	7.726
$R^{2}\;↑$	0.089	0.214
Pearson ↑	0.487 (p = 8.22× $10^{-6}$ )	0.584 (p = 3.06× $10^{-8}$ )
Spearman ↑	0.443 (p = 6.21× $10^{-5}$ )	0.535 (p = 6.41× $10^{-7}$ )

However, we do compare our model to published results on the NB-Bench test splits (Thermo-tm and Thermo-seq). These are external benchmarks used in the NbBench paper, and we outperform the reported NbBench results on both splits. As shown in Tables 6, 7, our model consistently outperforms the NbBench reported results, reducing MAE by over 20% and improving $R^{2}$ by approximately 25% across both external benchmark splits. Conversely, TemBERTure (Rodella et al., 2024) performs substantially below both baselines under reproduction, yielding negative $R^{2}$ values (−2.20 and −1.39) and Spearman correlations of 0.21 and 0.39 on the splits. We attribute this to a fundamental domain mismatch: TemBERTure was trained on TemBERTureDB, derived from the Meltome Atlas, a proteome-wide thermal proteome profiling dataset. The code for this study is publicly available at https://github.com/FairuzShadmaniShishir/NanobodyThermostabilityPrediction.

**TABLE 6:** Comparison with NbBench results on the Thermo-tm test split.

Model	MAE ↓	RMSE ↓	$R^{2}\;↑$	Spearman ↑
NbBench (reported)	6.83	8.46	0.36	0.59
TemBERTure (reproduced)	14.58	17.0	−2.20	0.21
NbBayesLM (proposed)	4.99	6.32	0.56	0.78

**TABLE 7:** Comparison with NbBench results on the Thermo-seq test split.

Model	MAE ↓	RMSE ↓	$R^{2}\;↑$	Spearman ↑
NbBench (reported)	6.53	8.49	0.45	0.58
TemBERTure (reproduced)	14.10	16.21	−1.39	0.39
NbBayesLM (proposed)	5.20	6.84	0.57	0.74

### 4.7 Ablation study and additional analyses

This section addresses further experiments to the (i) matched-input ablations that isolate the value of variational Bayesian inference beyond deterministic neural predictors, (ii) feature-fusion ablations that quantify the contribution of physicochemical descriptors beyond PLM embeddings, and (iii) uncertainty *calibration* and *utility* analyses that justify epistemic and aleatoric decomposition for nanobody thermostability prediction.

#### 4.7.1 Ablation protocol

All ablations use the *same* train/validation/test split and identical preprocessing. We report MAE (↓) , RMSE (↓) , and $R^{2}$ (↑) on the held-out test set. To ensure fair comparison, baselines are evaluated under matched input spaces: (i) ESM-2 embedding only, and (ii) ESM-2 embedding concatenated with the full 17-dimensional physicochemical descriptor vector.

#### 4.7.2 Bayesian vs. deterministic neural baselines

Our fusion head implements variational Bayesian linear layers with diagonal Gaussian posteriors, trained by maximizing the ELBO with a KL regularizer (Blundell et al., 2015; Tran et al., 2019). Importantly, uncertainty is modeled in the fusion head, while the embedding processing branch can remain deterministic, which is a standard hybrid design for high-dimensional representations. To verify that observed performance gains are attributable to Bayesian formulation rather than an architectural advantage, we developed a capacity-matched deterministic baseline sharing identical depth (4 layers), input and output dimensions (384-in, two-out), and approximately equal parameter count. The deterministic fusion MLP (hidden widths 724 →362→ 180; 607,844 parameters) is configured with reduced parameters compared to Bayesian counterpart (723,972 parameters) corresponding to an approximate 16% reduced parameter, reflecting the fact that Bayesian layers model each weight with both a mean and variance term, thereby placing the Bayesian model at a slight capacity disadvantage. The physicochemical branch is approximately matched in parameter count, although the intermediate layer widths differ slightly (Linear(17 → 64 → 256 → 128) vs. bayesian Linear(17 → 64 → 128 → 128)), while the ESM processor (789,760 params) and multi-head attention block (263,168 params) are strictly shared across both architectures. Total trainable parameter counts are 1,828,902 (Bayesian) *versus* 1,711,622 (deterministic), a 6.8% difference favouring the deterministic baseline.

Any performance difference between the two models therefore isolates the contribution of the probabilistic formulation itself, specifically, weight-distribution learning *via* the reparameterisation trick, KL-divergence regularisation as an implicit weight prior, and heteroscedastic negative log-likelihood training, none of which are present in the deterministic baseline.

#### 4.7.3 Results

Table 8 reports deterministic neural baselines trained on the same inputs. The proposed Bayesian fusion achieves the best overall predictive performance (MAE =1.89° C, RMSE =2.69° C, $R^{2}=0.670$ ) compared to (a) a deterministic NN on ESM-2 only and (b) a deterministic NN on ESM-2 + physicochemical features. Notably, simply appending physicochemical features to a deterministic NN *degraded* performance, whereas the Bayesian fusion model integrates modalities more effectively.

**TABLE 8:** Matched-input ablations on the held-out test set.

Model (inputs)	MAE ↓	RMSE ↓	$R^{2}↑$
Deterministic NN (ESM-2)	1.957	2.716	0.650
Deterministic NN (ESM-2 + physchem-17)	2.057	2.845	0.633
NbBayesLM (proposed)	1.89	2.69	0.670

#### 4.7.4 Interpretation

These ablations isolate two effects: (i) the Bayesian fusion head improves predictive accuracy relative to point-estimate neural training even when the input space is identical, and (ii) naive concatenation of physicochemical descriptors is not automatically beneficial under data scarcity; a probabilistic fusion head with KL regularization appears to improve robustness and mitigate overconfident fitting.

#### 4.7.5 Feature-fusion ablations

We also benchmarked multiple PLM embeddings (e.g., ESM-2, ProtBERT, ESMFold) combined with classical regressors - XGBoost (Chen, 2016), Linear (Su et al., 2012), Lasso (Ranstam and Cook, 2018), and Ridge (McDonald, 2009), with and without physicochemical features. Across models, physicochemical descriptors provide complementary biochemical information (e.g., charge/hydropathy/aromaticity) that can improve performance in certain regimes; however, the deterministic NN results in Table 8 show that *how* the fusion is performed matters, and motivates our probabilistic fusion design.

#### 4.7.6 Uncertainty calibration and decomposition utility

##### 4.7.6.1 Calibration metrics

We quantify probabilistic calibration using Prediction Interval Coverage Probability (PICP) (Wang, 2008) at 68/95/99% intervals, sharpness (Gneiting et al., 2007) (average interval width; lower is sharper given good coverage), and negative log-likelihood (NLL) (Yao et al., 2019). Table 9 summarizes the added evaluation.

**TABLE 9:** Uncertainty calibration metrics for the proposed Bayesian fusion model on the held-out test set.

Metric	Value
PICP@68%	0.934
PICP@95%	0.988
PICP@99%	0.997
Sharpness	4.968
NLL	2.1659

**FIGURE 4:**
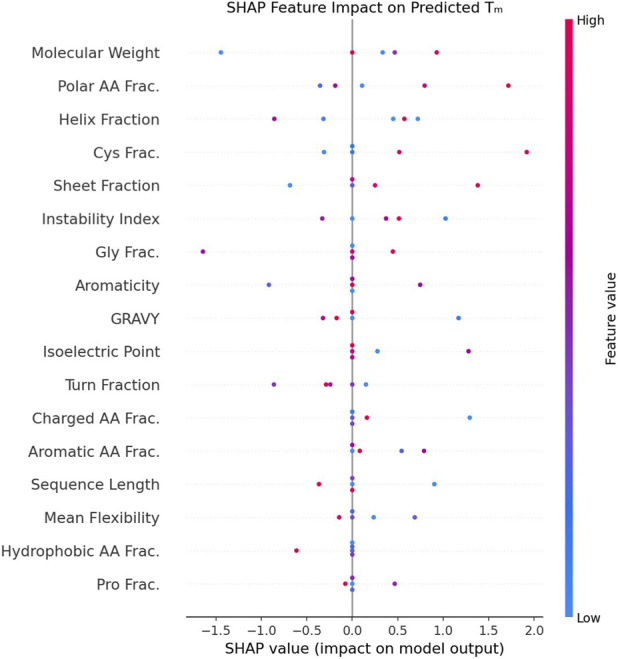
SHAP analysis for physicochemical features.

Figure 4 illustrates the PIT distribution for our probabilistic predictions. While ideal calibration would yield a uniform histogram, the observed inverted-U shape reflects conservative uncertainty estimates rather than over-confident ones. This behavior is particularly beneficial in protein engineering and drug discovery tasks, where cautious probabilistic estimates reduce the risk of falsely trusting incorrect predictions.

##### 4.7.6.2 Case study

Predictive uncertainty is decomposed into epistemic (model) and aleatoric (data) components to distinguish out-of-distribution (OoD) sequences from intrinsically ambiguous samples. Epistemic uncertainty is estimated *via* Monte Carlo sampling of the Bayesian neural network weights; aleatoric uncertainty is captured through the predicted variance term. Two representative sequences (sdab55, sdab219) (Huang and Lin, 2024) illustrate this distinction. We set the epistemic and aleatoric threshold of 4.01 and 3.89, respectively, based on 90% quantile. A high-epistemic sequence id sdab219 exibits ( $T_{m}=64.56\;{}^{\circ}$ C, with epistemic uncertainty of 4.33 ° C,butlowaleatoricuncertaintyof3.61° C). This sequence deviates from the training distribution through elevated sequence length and cysteine content, reflecting limited model familiarity and flagging a need for experimental validation for out-of-distribution (OoD) sequences. Conversely, a low-epistemic sequence id sdab55 with (low epistemic uncertainty of 3.48 ° C,highaleatoricuncertaintyof4.51° C) indicates confident learned representation alongside intrinsic target variability, likely arising from conflicting physicochemical signals or experimental noise. From a practitioner perspective, epistemic uncertainty can be used to prioritize sequences for experimental validation or dataset expansion, while aleatoric uncertainty can identify unreliable or noisy measurements that may require repeated experiments or careful interpretation.

## 5 Discussions

This study introduces a robust framework for predicting nanobody thermostability by combining protein language model (PLM) embeddings with physicochemical descriptors within a Bayesian neural network (BNN) architecture. Incorporating ESM-2 embeddings, which capture evolutionary conservation and latent structural information from large protein sequence datasets, substantially improves predictive accuracy when integrated with physicochemical features such as molecular weight, net charge, isoelectric point, GRAVY, instability index, and aliphatic index. Training on experimental data alone (630 sequences) yields the weakest performance (MAE 5.59±0.27 , $R^{2}\;0.53\pm 0.02$ ) alongside markedly elevated uncertainty (total: 134.04±15.88 ), consistent with severe data scarcity. Pseudo-labelled training (10,000 sequences) substantially recovers predictive performance (MAE 1.91±0.07 , $R^{2}\;0.62\pm 0.03$ ) and reduces total uncertainty to 26.50±1.20 , demonstrating that synthetic labels provide a meaningful supervisory signal despite the absence of direct experimental measurement. The combined dataset (10,630 sequences) achieves the best overall performance (MAE 1.89±0.08 , $R^{2}\;0.67\pm 0.02$ , total uncertainty 23.94±0.78 ), with incremental gains over pseudo-labelling alone suggesting that even a modest experimental anchor improves calibration and generalisation. Notably, the significant reduction in aleatoric uncertainty from 107.46±14.91 (experimental-only) to 12.76±0.66 (combined) reflects the stabilising effect of scale on intrinsic data noise estimates, and underscores the importance of dataset size for reliable uncertainty quantification. The Bayesian approach allows for principled uncertainty quantification, yielding epistemic uncertainty of 11.18 ± 0.16, aleatoric uncertainty of 12.76 ± 0.66, and total uncertainty of 23.94 ± 0.78, thereby modeling both limitations of the training data and inherent experimental noise. Our BNN, trained on 10,497 nanobody sequences with experimentally measured $T_{m}$ values, achieves a mean absolute error of 1.89 ° C and an $R^{2}$ of 0.67, surpassing previous methods.

Despite the substantial improvement afforded by pseudo-labelling, several limitations warrant acknowledgement. First, data scarcity remains a fundamental constraint: the experimental training set comprises only 630 sequences with directly measured $T_{m}$ values, which limits the diversity of thermostability phenotypes available for supervised learning and may reduce generalisation to structurally distinct nanobody families. Second, pseudo-label bias represents an inherent risk of the semi-supervised strategy. Pseudo-labels are generated by a model trained on the same limited experimental corpus, meaning systematic errors or biases in that model are propagated into the expanded dataset. If the base model underestimates or overestimates $T_{m}$ for particular sequence sub-families, this bias will be reinforced rather than corrected during combined training. Third, domain shift remains a concern when deploying the model on sequences that deviate substantially from the training distribution—including novel CDR topologies, non-standard framework regions, or nanobodies derived from camelid species underrepresented in the training data. In such cases, epistemic uncertainty estimates serve as a diagnostic signal, but cannot substitute for experimental validation. Taken together, these limitations suggest that model predictions should be interpreted with appropriate caution outside the training domain, and that continued curation of high-quality experimental $T_{m}$ data remains the most direct path to improved reliability. Futhermore, the heavy chain variable domain ( ${\text{V}}_{H}$ H) architecture characteristic of camelid nanobodies presents unique structural features, including extended complementarity-determining region 3 (CDR3) loops and disulfide bonding patterns, which may not be fully captured by current protein language models trained on broader protein sequence databases. The reliance on ESM-2 embeddings, while leveraging state-of-the-art representation learning, inherently carries the inductive biases of the pretraining corpus (UniRef50/90). This may result in suboptimal representations for nanobody-specific sequence motifs or structural elements that are underrepresented in general protein databases. Moreover, the selected physicochemical descriptors, though grounded in established protein stability principles, represent a reduced-dimensionality approximation of the complex thermodynamic landscape governing protein folding and stability. Furthermore, the variational inference framework employed in Bayesian neural networks introduces computational overhead compared to maximum likelihood estimation in deterministic models. The need to sample from approximate posterior distributions during both training and inference phases increases computational complexity by approximately two to three fold compared to standard neural networks. This computational burden may pose challenges for large-scale deployment or real-time applications, particularly in resource-constrained environments. Another limitation remains data scarcity, which could be mitigated through active learning–guided experimental characterization and the inclusion of synthetic sequences, provided rigorous validation ensures generalizability. Progress also depends on standardized evaluation protocols and benchmarks that enable robust comparisons across datasets, nanobody families, and experimental conditions.

### 5.1 Future directions

This study highlights several opportunities for advancing nanobody thermostability prediction. Incorporating three-dimensional structural information from AlphaFold2 or molecular dynamics could reveal spatial interactions critical to stability. Nanobody-specific protein language models trained on curated ${\text{V}}_{H}$ H datasets would yield more representative embeddings, while advanced variational inference methods (e.g., normalizing flows (Rezende and Mohamed, 2015), Hamiltonian Monte Carlo (Betancourt, 2017)) could improve uncertainty quantification with reduced computational cost. These innovations would support multitask frameworks predicting multiple biophysical properties (e.g., stability, affinity, expression) from shared representations.

## 6 Conclusion

We present a computational framework for predicting nanobody thermal stability that integrates protein language model (PLM) embeddings with physicochemical descriptors within a Bayesian neural network (BNN). Specifically, ESM-2 sequence representations are combined with molecular features such as hydrophobicity, cysteine frequency, and other stability-relevant descriptors to capture both high-level evolutionary semantics and low-level biochemical properties of nanobody sequences. This feature fusion enables the model to learn complementary information that is not accessible from sequence embeddings alone. The proposed model achieves accurate melting temperature $\left(T_{m}\right)$ prediction (MAE: 1.89 ° C, $R^{2}$ : 0.67) while simultaneously providing calibrated predictive uncertainty through Bayesian inference. Unlike deterministic PLM-based predictors, our framework quantifies confidence in each prediction, which is critical for experimental decision-making and prioritization in biologics development pipelines. The uncertainty estimates allow researchers to identify sequences that are both promising and reliable, thereby reducing dependence on costly and time-consuming wet-lab assays. Empirical comparisons demonstrate that the multi-modal fusion of PLM embeddings and physicochemical descriptors consistently outperforms models based solely on protein language representations. This highlights the importance of integrating domain-specific biochemical knowledge with deep sequence representations for stability prediction tasks. Although the current framework is limited by dataset scale and the computational cost associated with Bayesian inference, it provides an interpretable, probabilistic, and practically useful tool for nanobody engineering in therapeutic contexts. Future improvements are expected through the incorporation of structural features and more efficient approximate inference techniques, further enhancing predictive performance and scalability.

## Data Availability

The datasets and code for this study is publicly available at https://github.com/FairuzShadmaniShishir/NanobodyThermostabilityPrediction.
